# Comprehensive Evaluation of Anti-hyperglycemic Activity of Fractionated *Momordica charantia* Seed Extract in Alloxan-Induced Diabetic Rats

**DOI:** 10.1155/2012/293650

**Published:** 2012-12-20

**Authors:** Shailesh Kumar Choudhary, Gagan Chhabra, Dipali Sharma, Aruna Vashishta, Sujata Ohri, Aparna Dixit

**Affiliations:** ^1^Gene Regulation Laboratory, School of Biotechnology, Jawaharlal Nehru University, New Delhi 110067, India; ^2^Department of Zoology, University of Delhi, New Delhi 110 007, India

## Abstract

The present study evaluates anti-hyperglycemic activity of fractionated *Momordica charantia* (bitter gourd) seed extracts. Fasting blood glucose levels were evaluated before and after administration of different fractions of the seed extract. Among the three fractions tested, fraction Mc-3 (15 mg/kg b.wt.) showed the maximum anti-hyperglycemic activity and reduced blood glucose levels in experimental diabetic rats significantly. The activities of the key regulatory enzymes of glucose metabolism (hexokinase, pyruvate kinase, lactate dehydrogenase, and glucose-6-phosphate dehydrogenase) were determined in Mc-3-treated diabetic animals. Once-daily administration of the fraction Mc-3 for prolonged period of 18 days to the experimental diabetic animals did not result in any nephrotoxicity or hepatotoxicity as evident from insignificant changes in biochemical parameters indicative of liver and kidney functions. Further fractionation of the fraction Mc-3 by size exclusion chromatography resulted in a fraction, designated Mc-3.2, possessing anti-hyperglycemic activity. The fraction Mc-3.2 showed the presence of a predominant protein band of ~11 kDa on SDS-PAGE. Loss in anti-hyperglycemic activity of the Mc-3.2 upon protease treatment indicates the proteinaceous nature of the anti-hyperglycemic principles. Overall, the results suggest that *Momordica charantia* seeds contain an effective anti-hyperglycemic protein(s) which may find application in treatment of diabetes without evident toxic effects.

## 1. Introduction

Diabetes mellitus, a metabolic disorder, is a major global health concern with a projected rise in prevalence from 171 million in 2000 to 366 million in 2030 [1]. The disease is caused due to an insufficiency of insulin secretion, insulin action or both. While type 1 diabetes can be easily managed with regulated insulin administration, repeated administration of insulin prior to every meal is not desirable. Insulin treatment, if not managed properly, occasionally can result in severe hypoglycemia, a life-threatening situation. Continued administration of therapeutics such as sulfonylureas, biguanides, thiazolidinediones, and alpha glucosidase inhibitors used for the treatment of type 2 diabetes is also known to cause undesirable effects [2]. For this reason, there is an increased interest among diabetics for complementary and alternative medicine involving the use of traditional medicinal herbs and their products, and other dietary supplements [3].

*Momordica charantia* (Mc), commonly known as bitter gourd, is one of the most used plants for the treatment of diabetes and related conditions in traditional system of medicine world over [4–6]. The extracts from fruit pulp, leaves, and whole plants of Mc have been reported to exert anti-hyperglycemic activity in experimental diabetic animal models [7–10], or glucose-loaded rats [11, 12]. In addition, the plant has also been reported to possess other therapeutic activities like anti-tumor [13], anti-human immunodeficiency virus (HIV) [14], anti-ulcerogenic [15] and hypotriglyceridemic activities [16]. 

While a number of reports have been put forth demonstrating the anti-hyperglycemic activity of Mc, systematic studies to evaluate the effect of prolonged treatment with the fractionated seed extracts on the blood glucose levels together with acute toxicity studies have not been carried out.

Therefore, the present study focuses on the bioassay-guided fractionation of the acid-ethanolic extract of Mc seeds to identify the fraction that contains the active principle(s) responsible for the anti-diabetic activity. Further, the effect of long-term treatment with the active fraction on glycemic control and on the liver and kidney functions in diabetic rats was also investigated, as these studies would aid in evaluating the hepatotoxic and nephrotoxic effects, if any, of the prolonged treatment and proving to be nontoxic will facilitate the use of the active fraction(s) for diabetes management.

## 2. Materials and Methods

### 2.1. Plant Material and Animals

*Momordica charantia* seeds (“Pusa Vishesh” variety) were procured from Indian Agriculture Research Institute (IARI), Pusa, New Delhi [17] in large quantity to maintain the consistency of the stock for extract preparation. Chromatographic matrix Sephacryl S-100 high resolution (HR) was obtained from Pharmacia, Sweden. All the chemicals were of analytical grade and were procured from Sigma-Aldrich Chemical Co., USA, or Boehringer-Mannhiem, Germany, unless otherwise stated. 

Random bred male Wistar rats (12–14 weeks) were housed in the Small Animal Facility of the Jawaharlal Nehru University. The animals were provided with rat feed (Hindustan Lever Ltd, India) and water ad libitum. The use of animals was duly approved by the Institutional Animal Ethics Committee of the JNU, and the guidelines prescribed by the Institutional Animal Ethics Committee, JNU, New Delhi, were followed while handling animals. 

### 2.2. Seed Extract Preparation and Fractionation

Extraction of seeds was done essentially as described earlier [18] with minor modification. All extraction and purifications were performed at 4°C. Decorticated seeds were extracted in 10 volume (w/v) of 75% ice-cold acid ethanol containing 0.2 N HCl and 1 mM PMSF and incubated overnight (O/N) at −20°C to give rise to crude extract (Mc-C). This was then centrifuged at 20,000 ×g for 1 h to give rise to the pellet fraction (Mc-0) and the supernatant fraction (Mc-1). After removal of ethanol by speed-vac concentration, concentrated fraction Mc-1 was fractionated by differential salt precipitation using 0.1–1 M ammonium carbonate gradient (pH 7.0) followed by centrifugation at 20,000 ×g for 1 h. The insoluble and soluble fractions were designated as Mc-2 and Mc-3, respectively. Bioactivity of the fractions was measured at each step of purification. 

### 2.3. Induction of Diabetes in Rats

The male Wistar rats were made diabetic by using alloxan. Briefly, alloxan was administered i.p. after starving the animals for 36 hrs at a dose of 150 mg/kg body weight (b.wt.). Animals were stabilized for three days by insulin administration, 1-2 units per day for 2 days. Only those animals having blood glucose level more than 300 mg per 100 mL blood were selected for further analysis. 

### 2.4. Evaluation of Biological Activity of *Momordica charantia* Seed Fractions

The diabetic animals were grouped into experimental groups each containing minimum 5 rats. The doses of different fractions are expressed in terms of their protein content. Different groups were treated with different Mc seed fractions (15 mg/kg b.wt.). Diabetic animals treated with saline (vehicle) were included in the study as negative control. A group of diabetic animals treated with protamine zinc insulin (10 IU/kg b.wt., s.c., Boots Pharmaceuticals Ltd., India) served as standard reference control. A group of normal untreated non-diabetic animals was also included in the study. Fasting serum glucose (6 h fasting) was measured in blood drawn from the tail vein during the study period using glucose oxidase-peroxidase method [19] or a glucometer (Accu-chek). 

### 2.5. Dose and Time Kinetics of Anti-Hyperglycemic Effect of the Active Fraction

In order to determine the time by which the active fraction is able to bring about anti-hyperglycemic effect, a short-term (0–4.5 h) study was conducted by measuring the blood glucose levels within the indicated periods after administration. 

Short-term time kinetics of the active fraction in diabetic rats was determined with a dose of 15 mg/kg b.wt. administered intraperitoneally (i.p.) or insulin (10 IU/kg b.wt). The blood glucose levels were measured at different time intervals. Optimum dose of the active fraction was determined by administering the animals with different concentrations (5–25 mg/kg b.wt.) of the active fraction. Blood glucose levels were measured at 3 h after administration. 

### 2.6. Effect of the Anti-Hyperglycemic Fraction of *M. charantia* Seeds on Biochemical Parameters in Diabetic Rats

The rats were divided into different groups (five rats in each group): Group I—PBS-treated normal non-diabetic controls, Group II—PBS-treated diabetic rats, Group III—diabetic rats treated with 15 mg/kg b.wt. of the active fraction, and Group IV—the diabetic rats treated with protamine zinc insulin (10 IU/kg b.wt.). The first two groups of rats were given saline (vehicle) daily. The extract and insulin were administered at the selected dosage to Groups III and IV, respectively, every day for 18 days. Body weights of the untreated and treated animals were monitored throughout the study period. The rats were bled prior to sacrifice on the last day of the treatment by cervical dislocation. Serum was collected and subjected to biochemical analysis for hepatic function markers using assay kits from AutoZyme, India, by the method of Penttila et al. [20], and renal function markers, blood glucose, in automated chemical analyzer (Johnson & Johnson Vitros 250 chemistry analyzer). Total cholesterol was estimated according to the method of Zlatkis et al. [21]. Liver excised immediately after sacrifice was washed in chilled PBS and homogenized in 0.025 M sucrose prepared in 0.02 M triethanolamine buffer (TRA-HCl, pH 7.4). After centrifugation at 1,00,000 ×g, supernatant was used for the assay of different enzymes like glucokinase [22], pyruvate kinase [23], lactate dehydrogenase [24], glucose-6-phosphate dehydrogenase [25], and malic enzyme [26]. The glycogen content of the liver and skeletal muscle was estimated as described by Seifter et al. [27].

### 2.7. Gel Filtration Chromatography

The enriched fraction showing maximum biological activity was further fractionated by gel filtration chromatography using Sephacryl S-100 HR in 0.2 M NH_4_HCO_3_ (pH 7.2–7.4). The collected fractions were analyzed by SDS-PAGE and silver staining. The peak fractions designated as Mc-3.1, Mc-3.2, and Mc-3.3 were analyzed for anti-hyperglycemic activity using experimental diabetic rats as described before. 

### 2.8. Glucose Tolerance Test

The most active fraction obtained after gel filtration chromatography was assessed for its ability to induce glucose tolerance in normal Wistar rats. The rats were administered with 2 g/kg of glucose (i.p.) after 30 min of the test fraction at a dose of 15 mg/kg b.wt. (i.p.). Serum glucose levels were measured in the blood samples collected from the tail vein prior to glucose administration (considered as 0 h) and at different time intervals after glucose administration. 

### 2.9. Statistical Analysis

All the results were analyzed statistically using one-way ANOVA or Student's paired *t*-test for paired data of different levels of significance. All the results were expressed as mean ± S.D. *P* values less than 0.05 were considered significant.

## 3. Results

### 3.1. Anti-Hyperglycemic Potential of Seed Fractions of *Momordica charantia*

The crude acid ethanolic extract upon centrifugation gave rise to fractions Mc-0 (insoluble fraction) and Mc-1. Since fraction Mc-0 contained insoluble material, it was not administered in the animals. The fraction Mc-1 and the fractions Mc-2 and Mc-3 derived from Mc-1 were tested for their anti-hyperglycemic potential in experimental diabetic rats. As evident, fraction Mc-1 at the tested dose (15 mg/kg b.wt.) was able to lower blood glucose levels (Table 1). After having established that fraction Mc-1 possessed the anti-hyperglycemic activity, its further fractionation was carried out, which resulted in insoluble fraction (Mc-2) and supernatant fraction (Mc-3). Analysis of the anti-hyperglycemic activity of these fractions demonstrated the activity to be enriched in the fraction Mc-3. The fraction Mc-3 at a dose of 15 mg/kg b.wt. reduced the serum glucose levels by approximately 40% of the vehicle PBS-treated diabetic control animals by the 3rd day while only 25% reduction was brought about by equal amounts of fraction Mc-1. Fraction Mc-2 appeared to have no effect on glucose levels of diabetic animals and 5 out of 7 animals in the Mc-2 treated group died by the 3rd day (Table 1). Unlike, PBS-treated diabetic animals, serum glucose levels were maintained in the animals treated with insulin, without any further rise.

### 3.2. Dose and Time Kinetics of Anti-Hyperglycemic Effect of Fraction Mc-3

**Figure 1:**
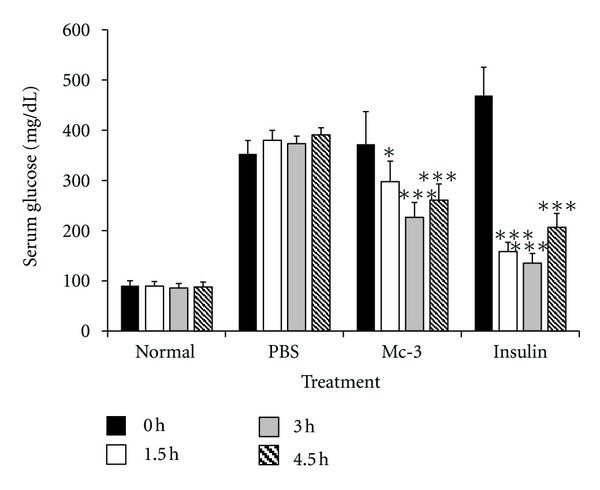
Short term anti-hyperglycemic effect of fraction Mc-3 of *M. charantia *seed extract: Diabetic animals were treated with fraction Mc-3 (15 mg/kg body wt., administered i.p.) or protamine zinc insulin (10 IU/kg b.wt.). Normal and control diabetic animals received equal volume of saline. Blood glucose levels were estimated at different time intervals post-intraperitoneal administration. Values are plotted as mean ± SD from at least five rats in each group. *P < 0.05; ***P < 0.001 compared with the respective group at 0 h.

Since fraction Mc-3 was found to possess enriched anti-hyperglycemic activity, it was desirable to study how quickly fraction Mc-3 could exert its anti-hyperglycemic effect after administration. Therefore, blood glucose levels of the Mc-3 treated animals were measured at different time after administration for a short period of 4.5 h. As shown in Figure 1, the fraction Mc-3 was able to bring down the blood glucose levels significantly by three hours and maintain the same even at 4.5 h after administration. Blood glucose levels were significantly reduced (~45%) in the diabetic animals treated with insulin within 1.5 h, which continued to remain lower during the experimental period. 

In order to assess the optimum concentration of Mc-3 that was able to bring about significant reduction in serum glucose levels of diabetic animals, the animals were administered with different concentrations of the fraction Mc-3 (5–25 mg/kg b.wt.) and the blood glucose levels were determined at 3 hr after administration (the time point determined earlier for visualizing the effect). It was observed that Mc-3 showed an increased reduction in blood glucose levels till 15 mg/kg b.wt. No further reduction in the blood glucose levels was observed when the animals were treated with a higher concentration (20 mg/kg b.wt.) of fraction Mc-3 (Table 2). 

### 3.3. Effect of Long-Term Fraction Mc-3 Treatment of Diabetic Animals

In order to assess the long-term effect of fraction Mc-3, the diabetic animals were maintained on fraction Mc-3 for a period of 18 days in order to assess if the continued administration of the active fraction Mc-3 had some toxic or undesirable effect on the liver and kidney functions. As expected, the blood glucose levels of the animals treated daily with Mc-3 were lower when compared to initial levels prior to the treatment (Table 3). A single daily injection of protamine zinc insulin was able to maintain fasting blood glucose levels in diabetic animals and resulted in slightly reduced serum glucose levels after one week of treatment. Unlike, the Mc-3 and insulin-treated group, the PBS-treated diabetic animals showed continued increase in blood glucose levels. 

After treatment, the serum activities of hepatic function markers: serum glutamic pyruvic transaminase (SGOT), serum glutamic oxaloacetic transaminase (SGPT), gamma-glutamyl transpeptidase (GGT), and the levels of renal function markers (urea and creatinine) were measured. Daily administration of insulin for 18 days resulted in significantly reduced levels of SGOT, SGPT, and GGT in comparison to PBS-treated diabetic controls. Similarly, the Mc-3-treatment of the diabetic animals for a period of 18 days also resulted in a significant reduction in the serum glutamic pyruvic transaminase (SGOT), serum glutamic oxaloacetic transaminase (SGPT), and gamma-glutamyl transpeptidase (GGT) when compared to PBS-treated control diabetic animals (Table 4). Likewise, treatment of diabetic animals with Mc-3 also resulted in a reduction (~20%) in serum cholesterol levels. Insulin-treated diabetic controls also showed reduced levels of total cholesterol. No significant change was observed in urea and creatinine levels, indicative of kidney function, in both the insulin as well as Mc-3-treated diabetic animals (Table 4) when compared to PBS-treated diabetic control animals. Mortality of the diabetic animals was also significantly reduced by administration of different fractions of *M. charantia* seed extracts. The Mc-3 treated animals had a 100% survival during the study period of three weeks with a normal behaviour, whereas the untreated control diabetic animals were lethargic, week and showed only about 60% survival by the end of three-week period. 

### 3.4. Effect of Fraction Mc-3 on Tissue Glycogen and Enzymes of Glucose Metabolism

**Figure 2:**
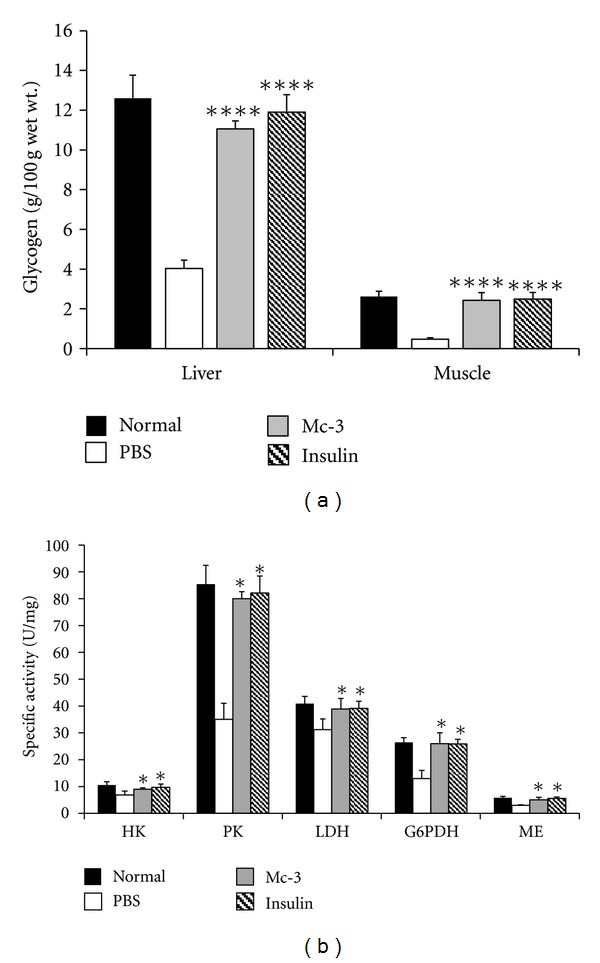
Effect of fraction Mc-3 of *M. charantia* seed extract on (a) liver and muscle glycogen and (b) activities of certain enzymes of glucose metabolism in diabetic rat liver. PBS-treated and protamine zinc insulin-treated (10 IU/kg b.wt.) diabetic animals were taken as negative and positive controls, respectively. Values are plotted as mean ± SD from at least five rats in each group. *P < 0.05; **P < 0.01; ****P < 0.0001 compared with the PBS-treated diabetic control group.

In order to determine if the reduction in serum glucose levels by Mc-3 treatment is due to increased glucose utilization, liver and muscle glycogen levels were measured. A significant reduction in both the liver and muscle glycogen levels was observed in the diabetic animals when compared to normal controls. Mc-3 treatment resulted in an increase of ~63% and ~80% in glycogen content of liver and muscle when compared to the PBS-treated diabetic animals (Figure 2(a)). Both the liver and muscle glycogen levels were also found to be elevated to almost normal levels in the diabetic animals treated with insulin.

The effect of Mc-3 treatment on key enzymes of glucose utilization in the livers of treated animals was also evaluated. To understand the possible mechanism of action, the diabetic animals were administered with either fraction Mc-3 or same volume of saline and maintained for 3 days on respective treatment. On day 4, the animals were injected with the test samples and were sacrificed after 3 hr of injection. Activities of the various regulatory enzymes were estimated in the livers of the control (saline treated) and Mc-3-treated diabetic animals (Figure 2(b)).

Control diabetic animals showed reduced levels of all the enzymes when compared to normal controls. It was observed that fraction Mc-3 resulted in a significant increase (~2 fold) both in pyruvate kinase (PK) and glucose-6-phosphate dehydrogenase (G6PDH) activities, whereas only ~1.3 and ~1.6 fold increase in the specific activity of hexokinase (HK) and malic enzyme (ME) was noted. However, no significant change in lactate dehydrogenase (LDH) activity was observed. Insulin-treatment also resulted in an increase in the activities of all the enzymes and restored their levels to that observed in normal non-diabetic animals. 

### 3.5. Gel Filtration Chromatography of Fraction Mc-3

**Figure 3:**
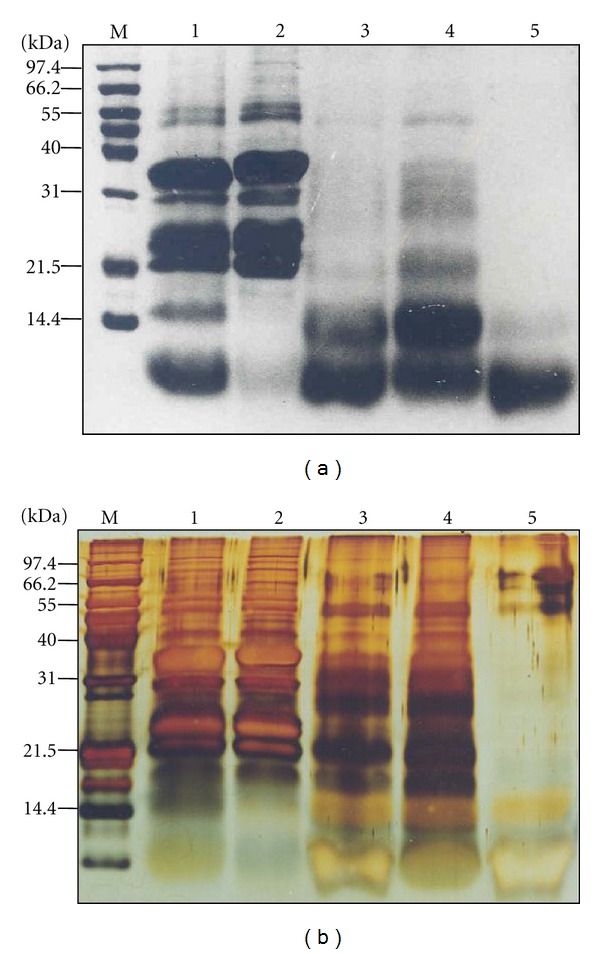
SDS-PAGE analysis of different fractions of *M. charantia* seed extract under reducing condition. (a) Crude extract and subsequent fractions of *M. charantia* seed extract were electrophoresed on 15% SDS-PAGE stained with Coomassie brilliant blue CBB-R250. M indicates protein molecular weight markers in kilodaltons. The gel was stained with CBB-R250. (b) Identical gel of different *M. charantia* fractions as shown in panel (a) stained with silver stain. M indicates protein molecular weight markers. Lanes 1–5 represent parent fractions Crude *M. charantia* extract (Mc-C), insoluble fraction Mc-0, soluble fraction Mc-1 and fractions Mc-2 and Mc-3 derived from fraction Mc-1 (45 *μ*g each), respectively, in both the panels.

**Figure 4:**
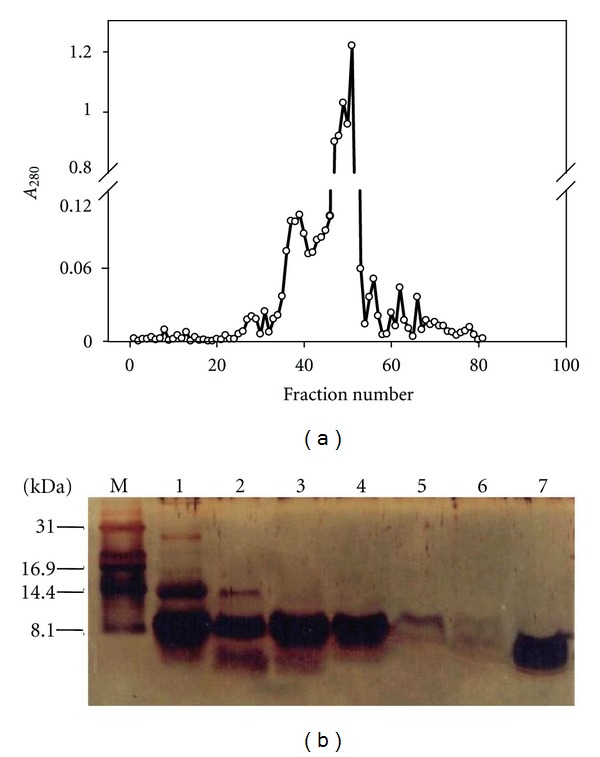
(a) Gel filtration chromatography of fraction Mc-3 of *M. charantia*: fraction Mc-3 was subjected to fractionation on a SephacrylR S-100 HR (2.5 × 80 cm) column. The figure shows a plot between fraction numbers and corresponding absorbance at 280 nm. (b) Fraction Mc-3 (15 *μ*g, lane 1) and its fractions obtained from gel filtration chromatography were analyzed on reducing SDS-PAGE using Tris-Tricine buffer system. Lanes 2–7 represent fractions 31, 35, 39, 43, 47, and 51, respectively (indicated in panel (a)). Lane M represents migration of protein molecular weight markers.

Earlier reports have indicated that the Mc fruit/seeds contain polypeptides that are capable of reducing blood glucose levels [28, 29]. Loss of activity upon heat treatment and protease K treatment of fraction Mc-3 confirmed the principle(s) to be of proteinaceous in nature (data not shown). Since the anti-hyperglycemic activity present in Mc-3 was found to be proteinaceous in nature, SDS-PAGE analysis of the fraction Mc-3 was carried out together with other parent fractions. The fraction Mc-3 appeared to be fairly pure, mainly consisting of protein band ranging from 8 to 14 kDa on a commassie stained SDS-PAGE (Figure 3(a)), and silver staining of the same revealed the fraction Mc-3 to contain many other protein bands (Figure 3(b)). Also, the band that appeared as a thick band (8–14 kDa) showed the presence of multiple protein bands when analyzed on a peptide gel in Tris-Tricine buffer. Therefore, gel filtration chromatography was carried out to further fractionate the proteins and to determine which of these possessed the anti-hyperglycemic activity. The chromatogram showing the elution profile of different proteins is shown in Figure 4(a). Peptide gel analysis of different peak fractions on SDS-PAGE using Tris-Tricine buffer system revealed that substantial purification of proteins from each other was achieved after gel filtration chromatography (Figure 4(b)). 

### 3.6. Fraction Mc-3.2 of Fraction Mc-3 Possesses Hypoglycemic Activity

**Figure 5:**
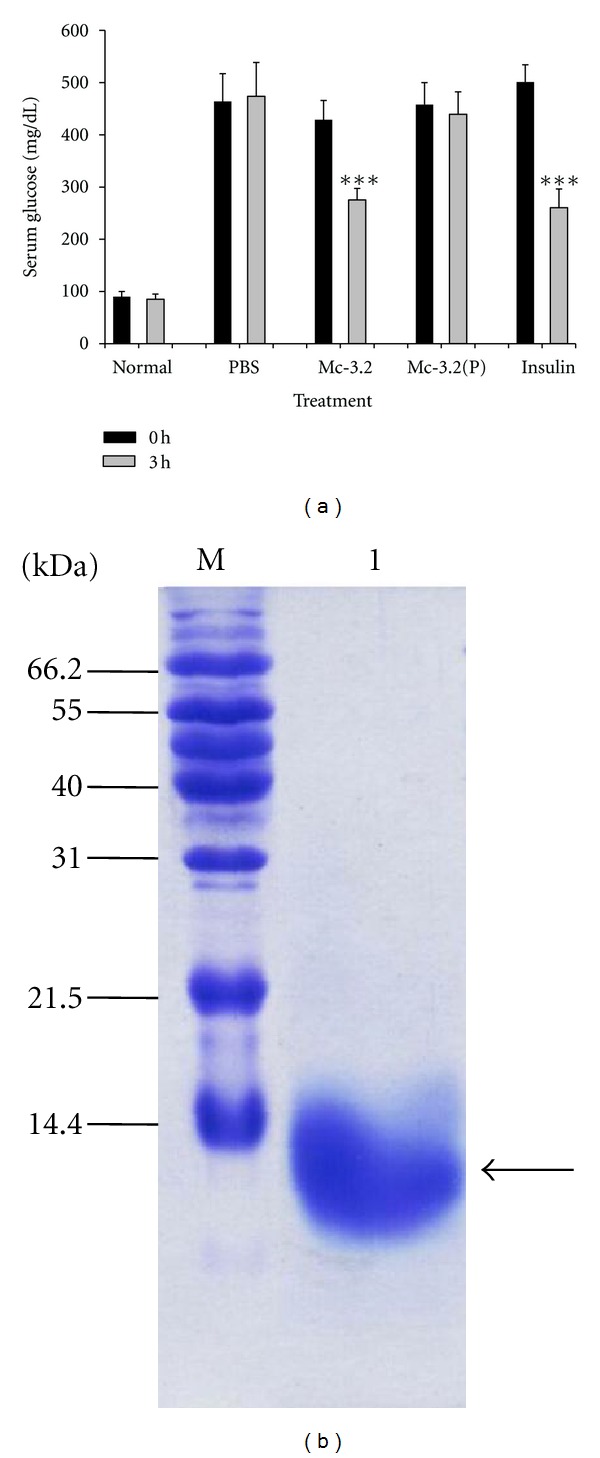
(a) Determination of the anti-hyperglycemic effect and putative nature of fraction Mc-3.2 of *M. charantia*: Diabetic animals were treated with fraction Mc-3.2, or protease-treated fraction Mc-3.2 (Mc-3.2(P), 15 mg/kg  b.wt. each)). Diabetic animals treated with equal volume of saline were included as negative controls whereas diabetic animals treated with protamine zinc insulin (10 IU/kg b.wt.) were taken as positive controls. Fasting serum glucose levels were estimated at 3 h postintraperitoneal administration. Values are plotted as mean ± SD from at least five rats in each group. *** indicates significance at *P* < 0.001 compared with the respective group at 0 h. (b) SDS-PAGE analysis of active peak fraction Mc-3.2 under non-reducing condition. M indicates protein molecular weight markers and lane 1 represents fraction Mc-3.2 (15 *μ*g).

In order to determine which of the major peak fractions consisted of the anti-hyperglycemic activity, these fractions were dialyzed and tested for their anti-hyperglycemic potential in diabetic animals. Fractions corresponding to different peaks were designated as fractions Mc-3.1 (fractions 31-32), Mc-3.2 (fractions 35–40), Mc-3.3 (fractions 43–46), Mc-3.4 (fractions 47–49), and Mc-3.5 (fractions 50–53). Fraction Mc-3.1 could not be evaluated for its anti-hyperglycemic activity due to small amounts. Of the remaining fractions, only fraction Mc-3.2 resulted in significant decrease (~40%) in blood glucose levels (Figure 5(a)). The other peak fractions did not bring about any significant reduction in blood glucose levels (data not shown). Protease treatment of the fraction Mc-3.2 resulted in the loss of its anti-hyperglycemic activity, confirming the previous results obtained with fraction Mc-3, that is the anti-hyperglycemic principle(s) present in *M. charantia *seeds was proteinaceous in nature. SDS-PAGE analysis of the active peak fraction Mc-3.2 showed that this consisted of dominant protein(s) of ~10–12 kDa on a non-reducing SDS-PAGE (Figure 5(b)). 

**Figure 6:**
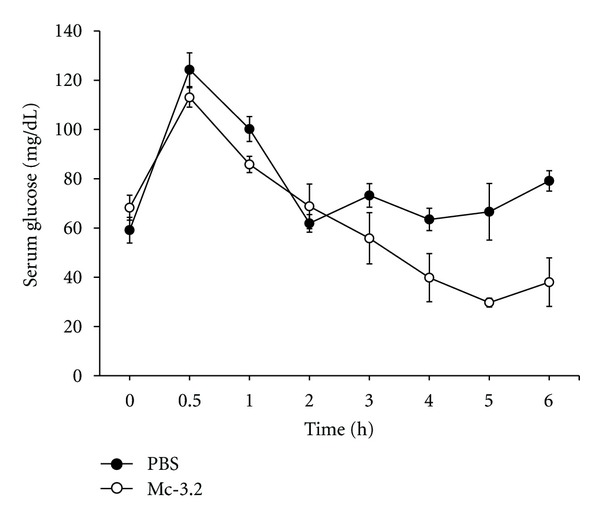
Glucose tolerance test (GTT) with fraction Mc-3.2 in normal rats. After glucose load of 2 g per kg b.wt., normal Wistar rats were treated with fraction Mc-3.2 (15 mg/kg body wt) and equal volume of PBS (control). Changes in serum glucose levels were determined at different time intervals. Values are plotted as mean ± SD from at least five rats in each group.

To check the ability of fraction Mc-3.2 to maintain normoglycemia in normal animals, glucose tolerance test was performed. Administration of Mc-3.2 resulted in faster clearance of blood glucose levels in normal rats when compared to PBS-treated controls, without causing hypoglycemia (Figure 6). At this dose, all animals were conscious with normal behavior and physical activity. Thus the results show that Mc-3.2 at 15 mg/kg b.wt. is not toxic to the animals, resulted in faster and consistence clearance of blood glucose after glucose load without causing hypoglycemia.

## 4. Discussion

Hyperglycemia associated with diabetes mellitus can be controlled by diet management, exercise, oral hypoglycemic agents, and insulin therapy. Both insulin therapy and oral hypoglycemic agents have their own side effects and adaptation problem. The secondary complications of diabetes that appear with lapse of time are actually the major cause of morbidity and mortality. Therefore, development of new approaches for treatment of diabetes that can reduce blood sugar level with better adaptation is desirable.

The evaluation of plants and especially of their active principles is a logical way of searching for new drugs to treat the disease. However, the presence of undesirable hyperglycemic substances along with the hypoglycemic components in *Dioscorea dumetorum* extracts has been reported [30]. Thus, thes search for a new therapeutic derived from plant for the treatment of diabetes will depend on the proper processing of the plant extracts in order to obtain the desired effect. The detailed toxicological studies both on crude plant extract as well as on the purified substances are also necessary. 

*Momordica charantia *has been used in indigenous system of medicine since a long time. Several reports have been put forth suggesting different parts of the plant to possess hypoglycemic activity. These have been found to be effective in chemically (alloxan and STZ) induced experimental diabetic rats [7–10].

Many small molecular weight proteins, polypeptides with hypoglycemic and/or anti-hyperglycemic activities have been isolated from *Momordica charantia *[31–33]. Other peptide and protein molecules isolated from *M. charantia* referred to as Charantin and p-insulin have been reported to lower fasting blood sugar (~25–40%) in experimental diabetic animals [28, 34]. In the present study, fraction Mc-3 obtained from the acid-ethanol extract of the *M. charantia *seeds has resulted in ~40% reduction in blood glucose levels within 3 h of treatment with an onset of reduction within 1 h of treatment. The anti-hyperglycemic effect brought about by the Mc-3 fraction of *M. charantia* seeds was comparable to that observed with insulin treatment of the diabetic animals. Our observations in the insulin-treated animals are in agreement with the earlier reports where in a sharp decrease in the fasting serum glucose levels was observed in protamine zinc insulin within two hours [35]. Like earlier studies, prolonged treatment of diabetic animals with protamine zinc insulin was able to maintain plasma glucose levels [36, 37].

The anti-hyperglycemic activity of the fraction Mc-3 at a much lower dose (15 mg/kg b.wt.) is significantly higher than that observed with acid-ethanol extract (fraction Mc-1) or other fraction Mc-2 derived from Mc-1. It appears that fraction Mc-2 consists of some toxic compounds that caused skin lesion and necrosis at the site of injection. Over a period of 18 days treatment daily, the blood glucose levels of fraction Mc-3-treated diabetic rats were significantly reduced, a desirable criterion for any potential anti-diabetic agent. Also, no hypoglycemic condition was observed in the treated animals. The fraction Mc-3 was effective at much lower concentration (15 mg/kg b.wt.) when compared to that of the crude ethanolic extracts of other plants which were found to be effective in the range of 100–500 mg/kg b.wt. in diabetic rats [38–40]. Thus, fraction Mc-3 can work as an effective anti-diabetic agent as it normalized the blood sugar maintenance function, without causing hypoglycemia.

Hyperglycemic condition due to partial or total lack of insulin arises because of disturbances in glucose metabolism caused by a decrease in several key enzymes of glycolysis, namely, glucokinase, phosphofructokinase, and pyruvate kinase, thus resulting in impaired peripheral glucose utilization and augmented hepatic glucose production [41]. Also, chronic diabetes results in a decrease in liver weight due to enhanced catabolic processes such as glycogenolysis, lipolysis, and proteolysis [42]. An increase in tissue glycogen levels, the primary intracellular storage form of glucose upon treatment with fraction Mc-3 can directly be correlated with its anti-hyperglycemic activity. This could be due to stimulation of the glycogen synthesis and inhibition of glycogen phosphorylase [43, 44]. Similar results with an increase in the hepatic glycogen have also been reported with *S. cordatum *extract, capable of bringing down blood glucose levels [45]. Also, an increase in glucose utilization enzymes enhances peripheral glucose utilization and could contribute to the anti-hyperglycemic effect of fraction Mc-3. 

It is of interest to note that the treatment of diabetic animals with Mc-3 resulted in reduction in the serum levels of marker enzymes of liver function, namely, SGOT, SGPT, and GGT. An increase in the serum levels of hepatic function marker enzymes generally arises due to necrotized liver conditions caused by chronic diabetes [46, 47]. Further, the two transaminases, SGOT and SGPT, belong to gluconeogenesis pathway and their increased concentrations in serum in diabetic condition further increase serum glucose levels by converting noncarbohydrate sources in the blood to glucose. Thus a reduction in the levels of these marker enzymes by Mc-3 treatment suggests that fraction Mc-3 is able to alleviate liver damage caused by alloxan-induced diabetes. Also, no significant changes in the urea and creatinine indicate that continued Mc-3 treatment did not adversely affect kidney functions. 

Earlier reports on the anti-diabetic potential of *M. charantia* have employed extracts of whole fruit, pulp, and leaves in experimental diabetic animals of glucose-loaded rats [7–12]. The present study reports substantial purification of a proteinaceous anti-hyperglycemic principle(s) from fractionated ethanolic extracts of *M. charantia* seeds. The anti-hyperglycemic principle(s) is highly effective in bringing down blood glucose levels to near normoglycemia, at much lower concentrations without causing any adverse effect on liver and kidney function of the treated animals. Studies are in progress to further purify and characterize the active proteinaceous principle present in fraction Mc-3.2 of *M. charantia*, which will be helpful in understanding the molecular mechanisms by which anti-hyperglycemic effect is brought about.

## Figures and Tables

**Table 1:** Effect of different fractions of *M. charantia* seed extract on serum glucose levels (mg/dL) in diabetic rats.

Period (days) of treatment	0	1	3
Treatment			
Normal	83 ± 3.6	84.4 ± 4.03	83.4 ± 5.3
PBS	457 ± 72.9	581 ± 69.2	615 ± 98.5
Mc-1	453 ± 80.5	347 ± 86.2*	318 ± 82**
Mc-2	463 ± 41	432 ± 54.5	ND
Mc-3	445 ± 75.1	317 ± 65.0*	246 ± 22.5***
Insulin	394.8 ± 40.1	329.4 ± 23.8*	336.5 ± 29.9

The diabetic animals were treated with different fractions (Mc-1, Mc-2, and Mc-3) at a dose of 15 mg/kg b.wt. and protamine zinc insulin (10 IU/kg b.wt), once daily for 3 days. Control normal and control diabetic animals were treated with corresponding volume of PBS. The data represent mean ± S.D. Each group consisted of at least 5-6 animals. *P < 0.05; **P < 0.01; ***P < 0.001 compared with the respective group on day 0. ND indicates “not determined.”

**Table 2:** Determination of the optimum dose of fraction Mc-3 for anti-hyperglycemic activity in diabetic animals.

Period (hr) of treatment	0	3	Reduction %
Normal control PBS	90 ± 10	85 ± 10	5.5
Diabetic control PBS	328 ± 17.6	314 ± 17.1	4.26
Mc-3 (5 mg/kg b.wt.)	372 ± 74.1	287 ± 10^*^	22.84
Mc-3 (10 mg/kg b.wt.)	301 ± 27.5	200 ± 18.2***	33.55
Mc-3 (15 mg/kg b.wt.)	541 ± 68.5	305 ± 43.4***	43.6
Mc-3 (20 mg/kg b.wt.)	474 ± 78.9	278 ± 42.0***	41.3
Mc-3 (25 mg/kg b.wt.)	368 ± 32.1	226 ± 25.9***	38.6
Insulin (10 IU/kg b.wt.)	512 ± 39.4	288.5 ± 33.3***	44

Diabetic animals were treated with different doses of fraction Mc-3. Fasting serum glucose (mg/dL) was measured before and 3 h post-administration. PBS-treated normal and diabetic animals were included as controls. Protamine zinc insulin-treated animals were included as positive controls. The data represent mean ± S.D. Each group consisted of at least 5-6 animals. *P < 0.05; ***P < 0.001, compared with the respective group at 0 hr.

**Table 3:** Effect of prolonged Mc-3 treatment on serum glucose levels.

Period (days) of treatment	0	3	6	9	12	18
PBS-treated normal	83.4 ± 8.3	81 ± 6.4	83 ± 7.2	85 ± 7.7	87.2 ± 6.3	88 ± 7.6
PBS-treated diabetic	407 ± 73	491 ± 42	5.6 ± 47	467 ± 44	456 ± 67	543 ± 80
Mc-3-treated diabetic	367 ± 106	250 ± 98**	240 ± 68**	238 ± 77**	210 ± 55**	189 ± 66***
Insulin-treated diabetic	418.92 + 23	334.2 + 61**	346.8 + 70	285.4 + 46***	263.94 + 47***	255.33 + 58***

Diabetic animals (*n* = 6) were administered with Mc-3 (15 mg/kg b.wt.) in PBS or protamine zinc insulin (10 IU/kg b.wt.) once daily. Fasting serum glucose levels (mg/dL) were measured on the days indicated. Control diabetic animals (*n* = 10) were treated with equal volume of PBS. The data represent mean ± S.D. *P < 0.05; **P < 0.01; ***P < 0.001, compared with serum glucose levels on 0 day.

**Table 4:** Effect of prolonged Mc-3 treatment on biochemical parameters in diabetic rats.

Parameters	PBS-treated normal	PBS-treated diabetic	Mc-3-treated diabetic	Insulin-treated diabetic
Liver function				
SGOT (IU/L)	135 ± 20.29	213.6 ± 34.71	133.75 ± 22.83**	126.75 ± 20.2***
SGPT (IU/L)	110 ± 12.53	225 ± 2.5	132.00 ± 16***	150.33 ± 13.31**
GGT (IU/L)	45.4 ± 3.44	116.2 ± 5.11	59.75 ± 10.96****	61.0 ± 8.21****

Kidney function				
Urea (mg/dL)	31.65 ± 4.6	61 ± 18	63 ± 20	70.95 ± 20.15
Creatinine (mg/dL)	0.46 ± 0.10	0.36 ± 0.07	0.45 ± 0.05	0.35 ± 0.06

Total cholesterol (mg/dL)	60.6 ± 5.32	101.5 ± 4.44	70.8 ± 11.48**	72.4 ± 8.02***

Diabetic animals were treated with fraction Mc-3 (15 mg/kg b.wt.) or protamine zinc insulin (10 IU/kg b.wt.) once daily for 18 days. The serum was analyzed for biochemical parameters related with liver and liver and kidney function. The data represent mean ± S.D. The control animals received corresponding volume of PBS. Each group consisted of 5-6 animals each. **P < 0.01; ***P < 0.001; ****P < 0.0001 compared with the PBS-treated diabetic control group.

## Abbreviations

Mc: *Mo* *mo* *rd* *ic* *a* *charantia*
b.wt.: Body weight
G-6-PDH: Glucose-6-phosphate dehydrogenase
HK: Hexokinase
PK: Pyruvate kinase
LDH: Lactate dehydrogenase
GTT: Glucose tolerance test.
